# Association of mean platelet volume with echocardiographic findings in patients with severe rheumatic mitral stenosis

**DOI:** 10.15171/jcvtr.2019.17

**Published:** 2019-05-23

**Authors:** Naser Aslanabadi, Ahmad Separham, Leyla Valae Hiagh, Farid Karkon Shayan, Mehrnoush Toufan, Samad Ghaffari, Elgar Enamzadeh

**Affiliations:** ^1^Cardiovascular Research Center, Tabriz University of medical sciences, Tabriz, Iran; ^2^Students’ Research Committee, Tabriz University of Medical Science, Tabriz, Iran; ^3^Connective Tissue Diseases Research Center, Tabriz University of Medical Sciences, Tabriz, Iran

**Keywords:** Mean Platelet Volume, Echocardiography, Rheumatic Stenosis, Mitral Valve

## Abstract

***Introduction:*** Rheumatic heart disease (RHD) is a relatively common cause of mortality among patients in the developing countries, and pure mitral valve failure is the most common form of RHD. An increase in the mean platelet volume (MPV) is considered as an independent risk factor for many cardiovascular diseases. This study aimed to evaluate the association of MPV with echocardiographic findings in patients with severe rheumatic mitral stenosis.

***Methods:*** In a descriptive, analytical study, 100 patients with severe rheumatic mitral stenosis referred to Shahid Madani hospital of Tabriz University of Medical Sciences and 100 age & sex-matched healthy individuals were included the study. MPV and echocardiographic findings including Wilkins score, left ventricular ejection fraction (LVEF), pulmonary artery systolic pressure, and left atrial spontaneous echo contrast (LASEC) were evaluated in both groups.

***Results:*** MPV in the case group was 10.45±0.98 and in the control group was 9.88±0.83. MPV in the patient’s groups was significantly higher than the control group (*P *= 0.001). Also, MPV in patients with positive LASEC findings was 10.69 ± 1.01 and in patients with negative LASEC findings was 10.25 ± 0.91. The difference was found to be statistically significant (*P *= 0.028).

***Conclusion:*** Patients with rheumatic mitral stenosis has a higher MPV compared to the healthy individuals, and it is associated with LASEC sign seen in echocardiography.

## Introduction

Rheumatic heart disease (RHD) is still amongst the most common cardiovascular diseases in children and adolescents in the developing countries.^1,2^ Pure mitral valve failure is the most common form of RHD being affected in about 85% of cases.^3^

One of the complications of mitral stenosis (MS) is thromboembolism (TE), and various factors have been found to be associated with increased risk of TE in this regard.^4-6^ These factors include, but are not limited to, age, mitral valve surface size, atrial fibrillation (AF), low blood flow in the left atrium, and spontaneous echo contrast (SEC), i.e., swirling motion of the blood in the echocardiography of the left atrium. ^7^

Mean platelet volume (MPV) is a marker of platelet size and is useful in predicting platelet activity. The larger platelets are more active than the smaller ones. This leads to the production of more thromboxane A_2_ and beta thromboglobulin and increases the propensity to the prothrombotic state.^6^ Previous studies have shown that increased MPV increases the risk of atherothrombosis and thus cardiovascular events.^8^ Also, the relationship between MPV and some echocardiographic findings, including SEC, has been proposed. SEC is a risk factor for the formation of left ventricular thrombosis and systemic embolism from the heart.^9^

In this regard, Yavuz et al studied the relationship between MPV and vascular events in patients with rheumatic mitral stenosis (RMS) and showed that MPV was significantly higher in patients compared to the healthy controls. The study stated that these patients should undergo antiplatelet therapy.^10^ In another study, Varol et al showed that MPV was significantly higher in patients with RMS than the control group.^11^

Several studies have been carried out so far to establish a link between MPV variations and risk of TE in patients with RMS. However, a few studies have examined the association between MPV and echocardiographic findings in these patients. Therefore, considering the importance of TE events in the increased mortality and morbidity of patients with RMS, this study was conducted to evaluate the association between MPV and echocardiography findings in patients with severe RMS.

## Materials and Methods

Due to the absence of any previous study in this filed, a pilot study was carried out using 30 cases to determine the sample size. Accordingly, in a descriptive analytical study, 100 patients with severe RMS referred to the Department of Echocardiography at Shahid Madani hospital of Tabriz University of Medical Sciences (TUOMS) between were enrolled. This study was conducted for two years between April 2015 and April 2017.

The patients with severe RMS diagnosed via transesophageal echocardiography (TEE) who had written informed consent to participate in the study were included. On the other hand, patients under anti-platelet or anticoagulant therapy, and subjects with diabetes, hyperlipidemia, hypertension or coronary arterial disease were excluded from this study. Also, patients with liver, thyroid as well as kidney diseases, moderate mitral regurgitation or more, moderate aortic valve regurgitation or more, aortic stenosis, blood disorders, any history of cancer, connective tissue or inflammatory disease, and acute or chronic infection were not included in this study.

Based on the inclusion and exclusion criteria, 100 patients with severe RMS and 100 age and gender-matched healthy individuals were selected using convenience sampling and entered into the study. Demographic data of both groups including age and sex, past medical history, as well as drug history and NYHA FC were recorded in the relevant checklist. All subjects in the patient group underwent TEE and in the healthy group underwent transthoracic echocardiography, and the findings were recorded. These findings included Wilkins score, left atrial volume index (LAVI), left ventricular end-diastolic echo dimensions (LVEDD), left ventricular ejection fraction (LVEF), pulmonary artery systolic pressure, the pressure gradient across the mitral valve, mitral valve area, and left atrial spontaneous echo contrast (LASEC). Eyeballing estimation was used to estimate the LVEF. Then, 5 cc peripheral blood sampling was performed in both groups and sent to the central laboratory of Shahid Madani hospital, TUOMS for CBC testing and the results including MPV were recorded. In the patient’s group, the patients were divided into two groups with Wilkins score of less than or equal to 8 and greater than 8, and MPV and echocardiographic findings were compared between two groups.

The SPSS^TM^ software version 22 was used for all statistical analyses. The obtained data were expressed as the mean ± standard deviation (SD), frequency and percentage. The normal distribution of data was evaluated using Kolmogorov-Smirnov test. Chi-square test was used to compare the qualitative variables. Also, quantitative variables between the two groups were compared using independent *t* test or Mann-Whitney U test. In all comparisons, *P *≤ 0.05 was considered to be statistically significant. Using the conducted pilot study and the formula for determining the sample size of correlational studies (α = 0.05, 1-β = 80%, and the 95% confidence interval) the minimum initial sample size of 100 was obtained and included in the study.

## Results

Of the 100 patients studied, 23 (23.0%) were male and 77 (77.0%) were female. Also, in the control group, 27 (27.0%) patients were male, and 73 (73.0%) were female. The difference was statistically insignificant (*P* = 0.930). Mean age was 49.69 ± 13.61 years (ranging between 21 and 82 years) and 50.43±14.2 years (ranging between 25 and 82 years) in the case and control groups, respectively. There was no significant difference between the groups in age (*P* = 0.780).The mean duration of the disease in the patients under study was 37.58±30.64 months.

Results showed that 75 patients (75.0%) were NYHA FC I & II, 21 (21.0%) patients were NYHA FC III, and four patients (4.0%) were NYHA FC IV. Also, we found that 45 (45.0%) patients had AF rhythm. Ten patients (10.0%) had a history of previous TE events, of which 10 (10.0%) had a previous history of cerebrovascular attack and one (1.0%) had the peripheral arterial disease. We also found that 14 (14%) patients had a Wilkins score ≤8, and 86 (86%) had a score higher than 8.

We showed that MPV was significantly higher in the patients’ group than the control group (*P* = 0.001) (Table 1). Also, echocardiographic examination of the groups showed a considerably higher LAVI (*P* = 0.001) and right ventricular diameter (RVD) (*P* = 0.009), and lower LVEF (*P* = 0.001) in the RMS patients compared with healthy individuals. The difference for LVEDD was shown to be insignificant (Table 2). Table 3 shows the findings of echocardiography in the included RMS patients.

**Table 1:** Intergroup comparison showed a significantly higher MPV in patients with sever RMS

**Variable**	**Healthy individuals**	**Case group**	***P*** **value**
Hemoglobin	78.41 ± 4.06	98.21±1.66	0.001
Platelet (*10^3^)	71.33±50.8	34.21±60.9	0.010
MPV	88.9±0.83	54.01±0.98	0.001
PDW	26.21±1.71	79.31±2.38	0.001

MPV, Mean platelet volume; PDW, Platelet distribution width; RMS, rheumatic mitral valve stenosis.

**Table 2:** Comparison of the findings of echocardiography in the studied groups

**Variable**	**Healthy individuals**	**Case group**	***P*** **value**
LVEF	59.45 ± 2.97	77.25±5.50	0.001
LVEDD	62.54±5.14	50.44±8.03	0.209
LAVI	73.42±8.21	71.48±27.87	0.001
RVD (mm)	29.13±4.16	46.33±4.87	0.009

Analysis showed a statistically significant difference in all studied parameters between two groups except for LVEDD. LAVI, left atrial volume index; LVEDD, left ventricular end-diastolic echo dimensions; LVEF, left ventricular ejection fraction; RVD, right ventricular diameter.

**Table 3:** Findings of echocardiography in the included RMS patients

**Variable**	**Mean**	**Max.**	**Min.**
Mitral mean gradient (mmHg)	9.25±5.26	32.40	2.20
Mitral valve area (cm^2^)	0.92±0.19	1.40	0.40
Systolic PAP (mmHg)	46.0±24.5	135	51
Wilkin's Score	9.37±1.07	13	7

RMS, rheumatic mitral valve stenosis; PAP, pulmonary arterial blood pressure.

Based on the left atrial SEC (LASEC), the patients’ group was divided into two subgroups of LASEC positive (n = 45) and LASEC negative (n = 55). Analysis showed that MPV in LASEC positive patients was 10.69 ± 1.01 and in LASEC negative ones was 10.25±0.91. Further analysis found a significantly higher MPV in LASEC positive patients than others (*P* = 0.028).

Also, based on Wilkins score which shows the severity of MS, patients were further subdivided into those with Wilkins score of 8 or less than 8 (n = 14), and those with a score higher than 8 (n = 86). Analysis revealed that the laboratory and echocardiographic findings of these two subgroups had no significant difference (Table 4).

**Table 4:** Comparison of the echocardiographic and laboratory results of the patients under study based on the Wilkins score

**Variable**	**Wilkins score>8**	**Wilkins score≤8**	***P*** ** value**
Hemoglobin	98.21±1.72	38.21±1.29	0.876
Platelet (*10^3^)	27.41±62.61	53.89±49.47	0.283
MPV	15.01±0.98	2.44±14.15	0.114
PDW	51.41±2.44	2.44±14.15	0.075
LVEF	52.46±5.75	54.64±3.07	0.171
LVEDD	44.28±7.07	42.67±12.69	0.650
LAVI	84.70±27.02	81.00±33.55	0.700
RVD (mm)	33.63±4.97	33.71±4.42	0.956
Mitral mean gradient (mmHg)	9.30±4.79	8.93±7.79	0.865
Mitral valve area (cm^2^)	0.91±0.19	0.97±.020	0.241
Systolic PAP (mmHg)	46.91±22.83	40.00±34.09	0.491

Intergroup comparison showed no significant difference in the studied variables between these subgroups. MPV, Mean platelet volume; PDW, Platelet distribution width; LAVI, left atrial volume index; LVEDD, left ventricular end-diastolic echo dimensions; LVEF, left ventricular ejection fraction; RVD, right ventricular diameter.

There was no statistically significant correlation between MPV and systolic pulmonary artery pressure (PAP) (r = -0.157, *P*=0.124). Also, there was no significant correlation between MPV and Wilkins score (r = 0.010, *P* = 0.920).

**Figure 1:**
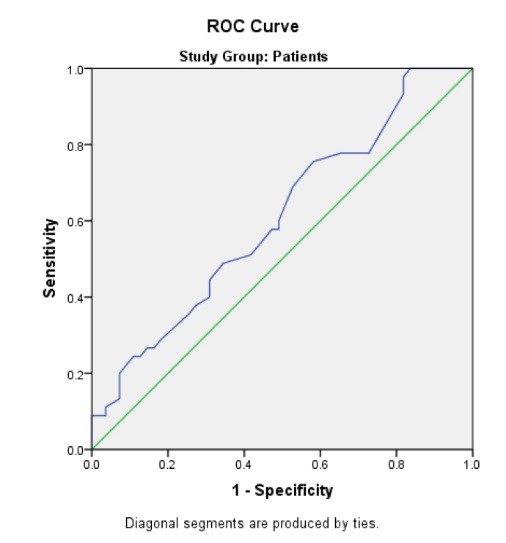
AUC-ROC for MVP.

AUC-ROC was found to be 0.607. Also, cut-off point for MPV in this curve was determined to be 9.95. At this point, the sensitivity and specificity were 0.756 and 0.418 with 95% CI (0.497-0.718), respectively (Figure 1).

## Discussion

MPV is an exact method to measure platelet size which reflects platelet function. It has been shown that larger platelets have higher hemostatical activity than their normal-size counterparts. This in turn escalates the risk for platelets aggregation (due to increased ADP secretion), thrombosis and TE.^12^ Increase MPV index has also been found to independently predict the risk for cardiovascular disease.^13^ A number of studies have revealed that severe RMS is associated with increased risk for platelets aggregation and thormobosis.^11,13^ However, in a majority of cases these data arise from retrospective investigations. Also, in several studies sample size is too small, and confounding factors affecting MPV obscure the findings. Intriguingly, the cut-off values resulting from these studies have not been authenticated in prospective studies.^14^ The need for a study which fulfills these demands is thus urgent.

Our study showed that patients with severe RMS had a significantly higher MPV than that of the control group. Similarly, Erdogan et al found that patients with RMS had a higher MPV than healthy controls, (9.05 ± 1.26 vs. 7.56 ± 0.74 fl, *P* < 0.001) This study also showed that platelets in RMS patients had higher activity than controls. Several factors could account for such an effect. First escalated shear stress against flow turbulence in damaged mitral valve leads to endothelial damage. Also, blood stasis induces platelet activation in the left atrium. As arms of Virchow triad, these factors lead to platelet activation and induction of thrombosis in RMS patients.^15^

Also, we found that MPV in LASEC positive patients was significantly higher than LASEC negative ones. In line with that, Akpek et al conducted a study to investigate the relationship between MPV and SEC in 232 patients with MS. The results showed that MPV in the SEC positive patients was significantly higher than SEC negative ones and. The MPV level increased with increasing SEC intensity. This study described MPV as an independent predictor of MS (OR = 2.365).^16^

Also, Ileri et al studied the MPV in patients with RMS and its association with echocardiographic findings. This study revealed that RMS patients had higher MPV than healthy subjects. Also, they showed that LASEC and severe mitral regurgitation were the most important predictors of increase in MPV in patients with RMS.^17^ In the present study, increase in MPV was associated with LASEC but did not show significant correlation with the severity of mitral regurgitation (Wilkins score). Similarly, Alyan et al showed that MPV in patients with chronic RMS was significantly higher than others.^18^ In this regard, Bayar et al also conducted a study to investigate the relationship between SEC and hematological factors in patients with RMS. MPV was found to be significantly higher in the LASEC-positive group (*P *< 0.001). The study found that MPV is an independent predictor of LASEC in patients with moderate to severe mitral valve stenosis.^19^

However, we found no correlation between MPV and Wilkins score as well as systolic PAP in these patients. However, Yardan et al examined the relationship between MPV and pulmonary arterial pressure in 150 patients with pulmonary embolism. This study found a direct correlation between MPV and systolic PAP and right ventricular diameter (RVD).^20^ The results of Kaya et al. study was in line with the findings of Yardan et al study.^21^

## Limitations

Most studies have reported an increase in MPV in patients with severe RMS. However, we found no difference between subgroups with high and low Wilkins score that could be due to several limitations that our study had; We could not take into account the effects of first, possible use of antiplatelet and anticoagulant drugs in some patients; second, difference in the duration of the disease between the subgroups; third, presence of AF rhythm in some patients which can affect the patient’s MPV; forth, significant difference in the number of patients with Wilkins score of less than or equal to eight compared to those with Wilkins score greater than 8, and last but not least, it can also be argued that due to the lack of association between MPV and echocardiography findings in RMS patients, other factors such as age, sex, and the inflammatory state of the body result in damage to the mitral valve and thromboembolic events, and MPV is not the only causative factor in this regard.

## Conclusion

Patients with rheumatic MS have a higher MPV compared to the healthy individuals, and it is associated with LASEC sign seen in echocardiography. However, this study showed no correlation between MPV and other echocardiographic findings in RMS patients. More studies with higher number of patients are needed to resolve the discrepancy seen between this study and others and to help better decision making.

## Competing interests

The authors declare that they have no conflict of interest.

## Ethical issues

This study was conducted after approval by the Ethics Committee of TUOMS. Informed written consent was obtained from the patients and healthy subjects or their first-degree relatives. All the information of the patients and healthy subjects was kept confidential, and their personal information was not mentioned anywhere. During the study, no additional therapeutic intervention was performed for the patients, and all costs were provided by the study implementor and sponsored by the vice chancellor of TUOMS.

## Acknowledgment

We are grateful to all our patients for their contribution and patience throughout this study. This paper is based on Leyla Valae High’s specialty dissertation submitted to Department of Cardiology, Tabriz University of Medical Sciences, Tabriz, Iran.
